# Risk of thromboembolism in cisplatin versus carboplatin-treated patients with lung cancer

**DOI:** 10.1371/journal.pone.0189410

**Published:** 2017-12-11

**Authors:** Eric S. Kim, Andrea M. Baran, Esther L. Mondo, Thomas D. Rodgers, Gradon C. Nielsen, David W. Dougherty, Kishan J. Pandya, David Q. Rich, Edwin van Wijngaarden

**Affiliations:** 1 Departments of Medicine / James P. Wilmot Cancer Center, University of Rochester, Rochester, New York, United States of America; 2 Biostatistics and Computational Biology, University of Rochester, Rochester, New York, United States of America; 3 Department of Public Health Science, University of Rochester, Rochester, New York, United States of America; Medical University Innsbruck, AUSTRIA

## Abstract

**Introduction:**

Carboplatin is widely used to treat lung cancer in the United States as an alternative to cisplatin. Several studies have demonstrated that cisplatin-based regimen is associated with a high frequency of thromboembolic complications. However, there has been limited investigation directly comparing the risk of thromboembolic events (TEEs) between cisplatin- and carboplatin-treated patients with lung cancer.

**Methods:**

All lung cancer patients treated with cisplatin or carboplatin at Wilmot Cancer Center, University of Rochester between 2011 and 2014 were included. Patient characteristics including exposure (cisplatin vs. carboplatin) and outcome (TEEs between the time of the first dose of cisplatin or carboplatin and 4 weeks after the last dose) were collected by reviewing electronic medical records. A Fisher’s exact test was used to compare the proportion of incident TEEs between cisplatin and carboplatin groups. The risk of TEE associated with carboplatin compared to cisplatin was assessed using multiple logistic regression.

**Results:**

Among 415 subjects, 317 patients (76.4%) received carboplatin and 98 (23.6%) patients received cisplatin. In the carboplatin group, 10.9% (33/302) of evaluable patients developed treatment-related TEEs vs. 14.7% (14/95) in the cisplatin group. There was no significant difference in the risk of developing TEEs between the two groups (*P* = 0.32). However, 15.2% of carboplatin-related TEEs were arterial thromboses compared to none in the cisplatin group.

**Conclusions:**

The incidence of carboplatin-related TEEs was high in lung cancer patients without significant difference in the risk of developing TEEs between cisplatin and carboplatin groups. Potential use of prophylactic anticoagulation in all platinum-treated patients should be further investigated.

## Introduction

Several lung cancer studies demonstrated that carboplatin and cisplatin offered similar overall survival, but carboplatin regimen was associated with a more favorable toxicity profile.[1–4] As such, carboplatin is being widely used throughout the United States. Santana-Davila and colleagues utilized the Veterans Affairs Central Cancer Registry to identify 4352 metastatic NSCLC patients and among those, 4061 (93%) patients received carboplatin.[4] This practice pattern favoring carboplatin use is seen across several medical oncology practices in the United States, including the Wilmot Cancer Center (WCC), University of Rochester (Rochester, NY).

Thromboembolic events (TEEs) significantly reduce the survival of patients with cancer[5, 6] and result in a substantial economic burden.[7] A large cohort study has shown that although cancer alone is associated with a 4.1-fold increase in the risk of thrombosis, the addition of chemotherapy enhances that risk to 6.5-fold.[8] Among chemotherapeutic agents, cisplatin-based regimens have been particularly associated with a wide range of thromboembolic complications.[9–11] A meta-analysis involving a total of 8216 patients with various advanced solid tumors from 38 randomized controlled trials reported that cisplatin is associated with a significant increase in the risk of TEEs in patients with advanced solid tumors when compared with non-cisplatin-based chemotherapy.[12] A retrospective study involving all cancer patients treated at Memorial Sloan-Kettering Cancer Center with cisplatin-based chemotherapy in 2008 reported 18.1% incidence of TEEs during active treatment with cisplatin or within 4 weeks of the last cisplatin dose.[13] A different retrospective study reported 8% incidence of TEEs in platinum-treated NSCLC population.[14] In this study, while the majority of the subjects received cisplatin, a subset of patients was treated with carboplatin and there was no apparent difference in incidence of TEEs between cisplatin (8%) and carboplatin (5%) groups. Furthermore, a prospective study involving 108 patients with stage III to IV non–small-cell lung cancer (NSCLC) treated with cisplatin and gemcitabine reported that 19 (17.6%) of 108 patients experienced a TEE and four of those 19 patients died as a result of the event.[15]

As stated earlier, carboplatin is being widely used in the United States as an alternative to cisplatin. To our knowledge, there has been limited study directly comparing the risk of developing TEEs between cisplatin- and carboplatin-treated groups in any malignant tumors including lung cancer. Therefore, we conducted a single-institution retrospective study to determine and compare the risk of developing TEEs between cisplatin and carboplatin groups in our recently-treated patients with lung cancer.

## Material and methods

### Patient population

This retrospective cohort study (approved by the Institutional Review Board of the University of Rochester, Rochester, NY–approved protocol #14098) included all adult lung cancer patients (all histologies) treated with first-line platinum-based chemotherapy (cisplatin or carboplatin) at the WCC from March 2011 to December 2014. The data were analyzed in a de-identified manner and the study was conducted in accordance with the Declaration of Helsinki. March 2011 was chosen to strictly include patients who received their first cycle of platinum-based chemotherapy after WCC implemented electronic chemotherapy orders (Epic Beacon, Verona, Wisconsin). All subjects included in the study also had to complete their entire platinum-based treatment at the WCC. For example, if patients received their first cycles of chemotherapy at the WCC but received subsequent cycles at a different infusion center, they were excluded from the study. This strict inclusion criterion was applied to the study to ensure the accuracy of all data collected, in particular, treatment dates, doses received and laboratory parameters.

### Data collection

Comprehensive treatment characteristics were collected, including chemotherapy regimens, treatment dates and total cumulative platinum dose (converted using molecular weight of platinum). Total cumulative dose of platinum was used in our analyses instead of the number of cycles (or doses) since there was a large heterogeneity in dosing schedules and dose reductions in our lung cancer population. To ensure accuracy of the database, independent chart reviews were conducted by two physicians and any discrepancies were resolved through a consensus discussion.

We defined TEEs as a deep venous thrombosis or arterial thrombosis that includes pulmonary embolism, cerebrovascular accident, and unstable angina/myocardial infarction (MI). Imaging studies (computed tomography, ultrasound, ventilation/perfusion scan, angiography and magnetic resonance imaging) from electronic medical records were reviewed to identify patients who experienced TEEs. Unstable angina/MI was based on the diagnosis captured in the electronic medical record. There was one patient who was diagnosed with a TEE at an outside facility with an available radiographic report confirming the event. All TEEs occurring after first platinum chemotherapy were captured. We classified TEEs as cisplatin or carboplatin-related if they were diagnosed between the time of the first dose of platinum and 4 weeks after the last dose. We reviewed all pre-treatment and post-treatment imaging modalities to ensure TEEs were new events not seen on previous imaging prior to initiation of chemotherapy.

### Covariates

Other baseline characteristics and covariates were collected using electronic medical records. The main clinical covariates we considered were the factors associated with increased risk of TEEs in patients with cancer.[16] The Eastern Cooperative Oncology Group (ECOG) performance status before starting treatment, personal history of prior TEEs, family history of TEEs, concurrent anticoagulation/antiplatelet therapy, renal function, Khorana variables (hemoglobin level, platelet and leukocyte counts, body mass index (BMI)), stage of cancer and smoking status were collected.

### Statistical analysis

The primary objective of the study was to compare the risk of developing TEEs between cisplatin and carboplatin groups. Baseline and treatment characteristics were compared between treatment groups using Fisher’s exact test for categorical variables and the nonparametric Wilcoxon rank-sum test for continuous variables. Univariate logistic regression was used to estimate the risk of TEE (and corresponding 95 percent confidence intervals [CI]) associated with carboplatin compared to cisplatin. Multivariate stepwise logistic regression was used to model the outcome (presence vs absence of treatment-related TEE) as a function of treatment (carboplatin vs cisplatin) and other candidate predictors. All statistical analyses were performed using SAS Version 9.4 (Cary, NC).

## Results

### Patient characteristics

Table 1 lists the patient characteristics of 415 patients with all types of lung cancer who received at least one dose of platinum chemotherapy at the WCC between March 1, 2011 and December 31, 2014. The most common reason for exclusion was receiving at least one cycle of chemotherapy prior to March 1, 2011 resulting in an inability to electronically verify the actual treatment date and dose administered. Among the 415 patients, there were 317 patients (76.4%) who were treated with carboplatin and 98 (23.6%) patients treated with cisplatin. There were 18 patients (4.3%) who received both cisplatin and carboplatin as part of their treatment regimen. The number of cycles of cisplatin versus carboplatin therapy was used to assign these patients to the appropriate group. The majority of the cases were NSCLC accounting for seventy five percent of the study population. Ninety one percent of patients had advanced stages (III and IV). TNM staging was also used for small cell lung cancer.

**Table 1:** Patient characteristics categorized by carboplatin and cisplatin groups.

Characteristic	Carboplatin	Cisplatin	P-value
Total Number of Patients (N = 415)	317	98	
Age (mean (SD))	65.1 (10.0)	59.4 (8.5)	<0.0001
Cumulative Platinum Dose (mean (SD))	1190.3 (699.1)	340.7 (150.0)	<0.0001
	Number of Patients (%)		
Gender			0.91
Male	168 (53.0%)	51 (52.0%)	
Female	149 (47.0%)	47 (48.0%)	
Ethnicity			0.65
African American	31 (9.8%)	8 (8.2%)	
Caucasian	280 (88.3%)	87 (88.8%)	
Other	6 (1.9%)	3 (3.1%)	
Diagnosis			0.001
Small Cell Lung Cancer	64 (20.1%)	38 (38.8%)	
Adenocarcinoma	158 (49.8%)	34 (34.7%)	
Squamous Cell Carcinoma	63 (19.9%)	22 (22.5%)	
Other	32 (10.1%)	4 (4.1%)	
Stage			0.0004
I	7 (2.2%)	2 (2.0%)	
II	18 (5.7%)	11 (11.2%)	
III	71 (22.4%)	39 (39.8%)	
IV	221 (69.7%)	46 (46.9%)	
ECOG Performance Status			0.001
0	48 (15.1%)	32 (32.7%)	
1	173 (54.6%)	42 (42.9%)	
2	79 (24.9%)	16 (16.3%)	
3	17 (5.4%)	8 (8.2%)	
Obesity (BMI ≥ 30 kg/m2)			0.39
No	255 (80.4%)	75 (76.5%)	
Yes	62 (19.6%)	23 (23.5%)	
Smoking			0.55
Current	158 (49.8%)	55 (56.1%)	
Former	129 (40.7%)	34 (34.7%)	
Never	30 (9.5%)	9 (9.2%)	
Prior TEE			1.00
No	253 (79.8%)	79 (80.6%)	
Yes	64 (20.2%)	19 (19.4%)	
Family history of TEE			0.08
No	224 (70.7%)	60 (61.2%)	
Yes	93 (29.3%)	38 (38.8%)	
Anticoagulants/Antiplatelet therapy			0.82
None	191 (60.3%)	64 (65.3%)	
LMWH	16 (5.0%)	3 (3.1%)	
Antiplatelet*	98 (30.9%)	28 (28.6%)	
Warfarin	12 (3.8%)	3 (3.1%)	
Khorana variables			
Platelet count ≥ 350,000/uL			0.79
No	237 (74.8%)	72 (73.5%)	
Yes	80 (25.2%)	26 (26.5%)	
Leukocyte count > 11,000/uL			0.79
	244 (77.0%)	74 (75.5%)	
Yes	73 (23.0%)	24 (24.5%)	
Hemoglobin < 10 g/dL or use of ESA			1.00
No	295 (93.1%)	92 (93.9%)	
Yes	22 (6.9%)	6 (6.1%)	
BMI ≥ 35 kg/m2			0.15
No	301 (95.0%)	89 (90.8%)	
Yes	16 (5.0%)	9 (9.25)	
Khorana risk score			0.73
1	175 (55.2%)	50 (52.0%)	
2	99 (30.9%)	32 (32.7%)	
3	39 (12.3%)	12 (12.2%)	
4	5 (1.6%)	3 (3.1%)	
Khorana risk group			0.74
Intermediate	273 (86.1%)	83 (84.7%)	
High	44 (13.9%)	15 (15.3%)	
Renal function			<0.0001
GFR ≥ 60	283 (89.3%)	98 (100%)	
GFR < 60	34 (10.7%)	0 (0%)	
Second agent			<0.0001
Etoposide	75 (23.7%)	66 (67.4%)	
Gemcitabine	19 (6.0%)	8 (8.2%)	
Pemetrexed	110 (34.7%)	19 (19.4%)	
Taxanes	106 (33.4%)	3 (3.1%)	
Other	7 (2.2%)	2 (2.0%)	

Abbreviations: SD, standard deviation; TEE, thromboembolic events; ECOG, Eastern Cooperative Oncology Group; BMI, Body Mass Index; LMWH, low molecular weight heparin; ESA, Erythropoiesis-stimulating agent; GFR, Glomerular Filtration Rate.

* Antiplatelet agents include asprin and clopidogrel.

Age, diagnosis, stage, renal function, cumulative platinum dose, ECOG performance status and second agent were significantly different between cisplatin and carboplatin groups. Khorana risk variables were well-balanced between the two groups.

### Carboplatin vs. cisplatin in the incidence and characteristics of TEEs

Of the 415 patients included in the study, 47 patients (11.3%) experienced a new TEE during platinum-based chemotherapy or within 4 weeks after their last treatment. Eighteen subjects who did not experience TEEs but without sufficient follow up information for at least 4 weeks from last treatment were not included in the main analysis. The number (%) of subjects experiencing TEEs in the carboplatin and cisplatin groups were 33 (10.9%) and 14 (14.3%), respectively (Table 2). There was no significant difference in the proportion of TEEs between the carboplatin and cisplatin groups (*P* = 0.36). There was also no statistically significant difference in the distribution of types of TEEs between the groups. However, there were 5 (15.2% of TEEs) cases of arterial thromboses in the carboplatin group compared to none in the cisplatin group. This represents 1.6% incidence of new arterial events in the carboplatin-treated population.

**Table 2:** Number (%) and types of thromboembolic events in carboplatin and cisplatin groups (N = 397).

	Carboplatin (N = 302)	Cisplatin (N = 95)	P-value
TEE diagnosed during treatment or within 4 weeks post last treatment			0.36
No	269 (89.1%)	81 (85.3%)	
Yes	33 (10.9%)	14 (14.7%)	
Symptomatic vs. incidental			0.062
Symptomatic	22 (66.7%)	5 (35.7%)	
Incidental	11 (33.3%)	9 (64.3%)	
Types of TEE			0.50
DVT	9 (27.3%)	6 (42.9%)	
PE	12 (36.3%)	5 (35.7%)	
DVT+PE	7 (21.2%)	3 (21.4%)	
Arterial thrombosis	5 (15.2%)	0 (0%)	
Types of DVT*			0.14
Peripheral	14 (87.5%)	5 (55.6%)	
Central	2 (12.5%)	4 (44.4%)	

Abbreviation: TEE, thromboembolic events; DVT, deep venous thrombosis; PE, pulmonary embolism.

*Peripheral cases included DVTs in the extremities. Central cases included TEEs in internal jugular, subclavian and superior mesenteric veins.

### Univariate and multivariate analysis of patient characteristics including exposure to carboplatin vs. cisplatin for the risk of TEEs

Logistic regression analysis was used to estimate the risk of TEEs associated with several patient characteristics including exposure to carboplatin vs. cisplatin (Table 3).

**Table 3:** Logistic regression analysis for thromboembolic events diagnosed during treatment or within 4 weeks post last treatment (N = 397).

Variable	OR	95% CI	P-value
Univariate analysis			
Carboplatin (vs cisplatin)	0.71	(0.36, 1.39)	0.32
Age	0.99	(0.96, 1.02)	0.42
Gender (Female vs Male)	1.21	(0.66, 2.23)	0.54
Ethnicity			0.06
AA vs Caucasian	0.19	(0.03, 1.41)	0.10
Other vs Caucasian	2.33	(0.46, 11.9)	0.31
Diagnosis			0.02
SCLC vs adenocarcinoma	2.14	(1.00, 4.59)	0.05
Squamous vs adenocarcinoma	1.25	(0.51, 3.08)	0.63
Other vs adenocarcinoma	3.95	(1.57, 9.94)	0.004
Stage			0.18
I vs IV	0.76	(0.09, 6.23)	0.80
II vs IV	0.23	(0.03, 1.77)	0.16
III vs IV	0.56	(0.26, 1.20)	0.13
Smoking			0.62
Current vs never	0.96	(0.34, 2.69)	0.94
Former vs never	0.71	(0.24, 2.07)	0.53
Prior TEE	1.37	(0.68, 2.78)	0.38
Family history of TEE	0.60	(0.30, 1.22)	0.16
Anticoagulants/antiplatelets			0.64
LMWH vs none	0.45	(0.06, 3.53)	0.45
Antiplatelet vs none	1.24	(0.65, 2.37)	0.51
Warfarin vs none	0.59	(0.07, 4.69)	0.62
ECOG performance status			0.31
1 vs 0	1.44	(0.56, 3.69)	0.44
2 vs 0	2.07	(0.75, 5.67)	0.16
3 vs 0	3.11	(0.78, 12.4)	0.11
Obesity (BMI ≥ 30 kg/m2)	1.67	(0.85, 3.28)	0.14
Khorana risk variables			
PLT ≥ 350,000/uL	1.38	(0.71, 2.66)	0.34
WBC > 11,000/uL	2.19	(1.14, 4.19)	0.02
HgB < 10g/dL or use of ESA	0.93	(0.27, 3.20)	0.90
BMI ≥ 35 kg/m2	0.63	(0.14, 2.77)	0.54
Khorana risk score			0.41
2 vs 1	1.43	(0.72, 2.86)	0.31
3 vs 1	2.06	(0.88, 4.82)	0.10
4 vs 1	1.34	(0.16, 11.43)	0.79
Khorana risk group	1.70	(0.79, 3.64)	0.17
(high vs intermediate)			
GFR (< 60 vs. ≥60)	0.49	(0.11, 2.13)	0.34
Cumulative platinum dose			
Carboplatin group	0.99	(0.94, 1.05)	0.81
Cisplatin group	1.11	(0.76, 1.62)	0.59
Second agent			0.10
Etoposide (vs taxanes)	1.05	(0.51, 2.17)	0.89
Gemcitabine (vs taxanes)	0.24	(0.03, 1.91)	0.18
Pemetrexed (vs taxanes)	0.47	(0.20, 1.12)	0.09
Other (vs taxanes)	2.00	(0.37, 10.85)	0.42
Multivariable analysis			
Carboplatin (vs cisplatin)	0.72	(0.36, 1.47)	0.37
Diagnosis			0.02
SCLC vs adenocarcinoma	2.07	(0.94, 4.53)	
Squamous vs adenocarcinoma	1.27	(0.51, 3.17)	
Other vs adenocarcinoma	4.00	(1.57, 10.19)	
WBC >11,000/uL	2.18	(1.12, 4.23)	0.02

Abbreviations: OR, odds ratio; CI, confidence interval; SCLC, small cell lung cancer; SCC, squamous cell carcinoma; TEE, thromboembolic events; LMWH, low molecular weight heparin; ECOG, Eastern Cooperative Oncology Group; ESA, Erythropoiesis-stimulating agent; PLT, platelet; WBC, white blood cell; HgB, hemoglobin; GFR, Glomerular Filtration Rate.

Univariately, diagnosis and WBC count (one of the Khorana risk variables) were significantly associated with the risk of developing TEEs. These variables remained independently associated with the risk of TEEs even after inclusion in a multivariate stepwise logistic regression model (Table 3).

There was no significant difference in the risk of treatment-related TEEs between the carboplatin and cisplatin groups (OR, 0.71; 95%CI, 0.36 to 1.39, *P* = 0.32). Similar to the univariate analysis, there was no significant difference in the risk of TEE between treatment groups (OR, 0.72; 95%CI, 0.36 to 1.47, *P* = 0.37) after adjusting for diagnosis and WBC count in a multivariate logistic regression model (Table 3). Even when we included in the multivariate analysis other variables known to be associated with TEEs such as age, stage, performance status and renal dysfunction, there was still no significant difference in the risk of TEEs between treatment groups (OR 0.61, 95% CI 0.27–1.34, *P* = 0.22)

As the cumulative platinum dose administered is markedly higher for carboplatin compared to cisplatin given differences in their pharmacokinetics properties, the potential influence of cumulative dose on the risk of developing TEEs was instead evaluated within each individual group. Cumulative platinum dose or the second agent given concurrently with cisplatin or carboplatin was not associated with the risk of TEEs (Table 3).

## Discussion

Based on our large retrospective analysis of 415 patients treated with platinum-based chemotherapy for lung cancer, we report a high incidence of TEEs in both the cisplatin-treated and carboplatin-treated groups. Our data not only confirm a high incidence of TEEs in cisplatin-treated patients as previously reported,[12, 13] but also demonstrate a new finding that 10.4% of lung cancer patients receiving carboplatin developed a new TEE either during treatment or within 4 weeks from their last carboplatin dose. There was no significant difference in the risks of developing TEEs between cisplatin and carboplatin. We also report a surprisingly high incidence of arterial thrombosis (15.2% of all TEEs) in patients receiving carboplatin while this could be due to the fact that subjects in carboplatin-treated group had more comorbidities associated with the risk of arterial events.

We believe that our findings are clinically significant as carboplatin is more frequently used in the United States, especially in advanced stages of lung cancer. Even at our institution, carboplatin was apparently a preferred agent over cisplatin which explains a larger number of subjects assigned to the carboplatin group. This likely reflects prescribing habits across various practices in the United States. As shown in Table 1, there was a statistically significant difference between cisplatin and carboplatin subgroups in the stage of cancer, renal function, performance status and diagnosis. This is expected as the majority of stage IV patients were treated with carboplatin consistent with previous report[4] and cisplatin was reserved more for curative stages. As expected, there was a higher percentage of cisplatin-treated patients with PS of 0 or GFR of greater than >60 mL / min compared to carboplatin-treated patients. Small cell lung cancer diagnosis represented the highest proportion of patients receiving cisplatin. In the univariate and multivariate analysis, diagnosis and WBC count influenced the risk of TEEs.

We collected information on several covariates which could influence the risk of developing TEEs in patients with cancer. Performance status which measures the degree of mobility has been widely recognized as a risk factor for TEEs.[15, 17] In our univariate analysis, performance status was not significantly associated with the risk of TEEs. This could be due to the subjective nature of determining accurate performance status, change in performance status over time and having to rely on provider’s clinic notes to collect data. Cumulative platinum dose or the second agent given concurrently with cisplatin or carboplatin was not associated with the risk of TEEs.

We collected Khorana risk variables[18] in all our subjects. Only WBC count was significantly associated with the risk of TEEs after multivariate analysis. Khorana risk score did not predict association with risk of TEEs in our population. This could be due to the fact that our study only involved a lung cancer population; every subject was assigned with a score of at least one which explains the lack of a low risk group in our study population. The causal relationship between WBC count and TEEs as well as the exact underlying mechanism remains unknown to our best knowledge. A different study also reported that elevated WBC, particularly neutrophils, is strongly associated with increased risk of venous thromboembolism and mortality in cancer patients receiving systemic chemotherapy.[19] However, it is certainly possible that TEEs can be associated with other conditions which themselves may cause leukocytosis. For example, Afzal et al. reported several causes for leukocytosis observed in patients with acute PE such as cancer, pneumonia, steroids, and atelectasis. Lung cancer being the study population, it is possible that majority of the subjects had other causes for leukocytosis.[20]

The major strengths of this study include use of well-characterized lung cancer patients in a single institution with comprehensive and accurate clinical information that was collected from the electronic medical record system by two independent physicians. The use of electronic medical records to collect data in a pre-defined modern chemotherapy period is a distinct advantage over other retrospective studies that evaluated subjects from a while back using paper charts. This study did not exclude patients based on age, performance status, medical comorbidities or socioeconomic status. As a result, we anticipate that our findings would be applicable to the general community population. Focusing only on the lung cancer population was an advantage distinguishing our study from others that evaluated all types of malignancy as rates of TEEs can vary significantly between cancer types.[16] For example, according to the California Cancer Registry, the rate of TEEs was 20% in patients with advanced pancreatic cancers, but only 0.9% and 2.8% in patients with advanced prostate and breast cancers, respectively.[6] Secondly, lung cancer is likely the only solid tumor with a large body of evidence demonstrating equivalence in efficacy between cisplatin and carboplatin.[1, 3, 12] This eliminates an important confounding element due to treatment effect influencing the risk of TEEs; a higher stage (more advanced cancer) is significantly associated with the risk of TEEs.[6, 17]

This is a single-institution retrospective cohort study that will require an independent, prospective validation to confirm our findings. One of the limitations of our analysis is inclusion of both arterial and venous thromboembolic events. While they may share similar risk factors, the pathophysiology for these events are thought to be different, potentially weakening association with certain variables. In addition, even though the study focused on lung cancer, it was limited by the fact that small cell lung cancer which may in itself be associated with high rate of TEEs was also included in the analysis. A small proportion of subjects (4.3%) received both cisplatin and carboplatin, and was assigned to the group with a greater number of cycles received. This limited definition was based on the assumption that more drug exposure is more likely to be associated with TEEs. Lack of significant relationship between several known risk factors of TEEs and TEEs in our analysis could be explained by a small number of events in some subgroups. Some variables such as hospitalization could not be reliably collected and therefore were not included in the analysis. Only a small subset of patients with stage I and II lung cancer receiving adjuvant platinum-based chemotherapy required hospitalization for resection. Furthermore, the majority of lung cancer patients with the exception of symptomatic SCLC patients received chemotherapy in the outpatient setting. We believe that the influence of these variables is similar in carboplatin and cisplatin groups with insignificant impact on our primary endpoint that is to simply make a comparison between the two groups. It is also possible that the increased risk of TEEs due to exposure to cisplatin or carboplatin could mitigate the added risk due to other variables.

In conclusion, this is the first study to our knowledge primarily comparing the risk of TEEs associated with carboplatin use compared to cisplatin use among lung cancer patients in the modern chemotherapy era. While there is a growing evidence for a high incidence of TEEs in patients receiving cisplatin-based therapy in all tumor types,[12, 13] our findings raise a significant clinical alarm that this high incidence of TEEs including arterial thrombosis is not specific to cisplatin alone but could be applicable to platinum agents in general. It would be important to educate our lung cancer patients regarding the increased risk of developing TEEs during platinum-based chemotherapy and to have low threshold for ordering diagnostic tests for TEEs with any clinical suspicion. Further study in identification of a high-risk group and development of optimal strategies to reduce the risk of TEEs is warranted.

## Supporting information

S1 Table Individual data for key variables. (PDF)
