# Correction: Risk of thromboembolism in cisplatin versus carboplatin-treated patients with lung cancer

**DOI:** 10.1371/journal.pone.0191454

**Published:** 2018-01-12

**Authors:** The PLOS Staff

The ninth author's name appears incorrectly in the XML of the article. The ninth author's name should be indexed as: van Wijngaarden E. The publisher apologizes for the error.
