# Supplementary material for: Risk of thromboembolism in cisplatin versus carboplatin-treated patients with lung cancer
Source: PLoS One. 2017 Dec 11;12(12):e0189410. doi: 10.1371/journal.pone.0189410 (PMC5724847; doi:10.1371/journal.pone.0189410)
Supplement: S1 Table — (PDF) [file pone.0189410.s001.pdf]

Cisplatin vs Carboplatin	Gender	Age	Ethnicity	Diagnosis	Stage	Smoking	ECOG PS	Creatinine	GFR	WBC	HgB	PLT	BMI	Thromboembolic Events (Yes=1 or No=0)
carboplatin	F	63	AA	other	IV	Former	1	1.45	44	6.7	9.2	179	26.0	0
carboplatin	F	56	Caucasian	adenocarcinoma	IV	Never	0	0.59	103	6	12.5	302	23.0	0
cisplatin	M	49	Caucasian	other	IV	Current	0	1.11	77	7.6	13.4	535	26.0	0
carboplatin	F	72	Caucasian	SCLC	IV	Current	1	1.4	37	2.6	10.5	339	19.5	0
carboplatin	F	62	Caucasian	SCLC	IV	Former	1	0.83	76	13.2	11.7	268	29.0	0
carboplatin	F	60	Caucasian	adenocarcinoma	IV	Never	1	0.63	98	4.2	10.5	155	29.0	1
carboplatin	M	72	Caucasian	Squamous	IV	Former	2	1.79	37	8.2	11.7	233	27.0	0
carboplatin	F	70	AA	other	III	Former	1	0.93	72	6.8	12	269	19.3	0
carboplatin	F	70	Caucasian	SCLC	IV	Current	1	1.03	55	9.2	12.2	353	27.2	0
carboplatin	F	64	Caucasian	SCLC	IV	Current	2	0.68	106	8.8	15.3	224	32.9	1
cisplatin	F	71	Caucasian	Squamous	III	Former	0	0.81	73	6.1	11.6	287	29.0	0
cisplatin	M	51	Caucasian	Squamous	III	Current	1	0.94	93	14.6	12.9	348	32.9	1
carboplatin	F	57	Caucasian	adenocarcinoma	III	Former	1	0.61	100	12.3	12.4	238	27.1	0
cisplatin	M	73	AA	SCLC	IV	Current	1	1.1	77	6.3	10.7	208	22.9	0
carboplatin	M	66	Caucasian	adenocarcinoma	IV	Current	1	1.3	56	6.5	14.9	281	28.9	0
carboplatin	F	79	Caucasian	other	IV	Former	1	0.85	65	8.9	9.7	558	30.3	0
cisplatin	F	59	Caucasian	Squamous	III	Current	1	0.92	68	8.7	13.8	287	28.2	0
carboplatin	M	69	Caucasian	squamous	III	former	0	0.83	90	10.7	11.3	394	29.5	0
carboplatin	F	71	Caucasian	SCLC	IV	Current	0	0.55	95	6.5	11.5	292	21.3	0
carboplatin	F	72	Caucasian	Squamous	III	Former	1	0.94	58	5.8	12.2	275	28.8	0
carboplatin	F	80	Caucasian	SCLC	IV	Former	2	0.7	82	6.4	13.8	331	17.9	0
cisplatin	M	51	Caucasian	SCLC	IV	Current	1	0.81	101	9.7	12.5	337	29.3	0
cisplatin	F	61	Caucasian	adenocarcinoma	III	Never	0	0.78	82	19.5	12.1	422	27.4	0
cisplatin	M	55	Caucasian	SCLC	IV	Current	1	0.71	105	5.6	11.2	322	24.5	1
carboplatin	F	47	Caucasian	SCLC	I	Current	1	0.7	103	6.9	14.2	339	23.6	0
carboplatin	F	64	Caucasian	adenocarcinoma	IV	Former	0	0.85	72	5.7	13.7	275	29.7	1
carboplatin	F	71	Caucasian	SCLC	IV	Never	1	0.75	80	9.2	16.1	226	22.1	1
carboplatin	M	81	Caucasian	squamous	III	former	1	1.06	65	5.9	13.7	179	29.3	0
carboplatin	M	65	Caucasian	other	IV	Former	1	0.85	91	10.1	13.1	324	33.8	0
carboplatin	M	61	Caucasian	adenocarcinoma	IV	Current	3	1.06	75	5.5	10.4	245	26.1	0
carboplatin	M	54	Caucasian	adenocarcinoma	III	Current	1	0.8	101	10.4	11	347	20.5	0
carboplatin	F	66	Caucasian	other	III	Former	1	0.99	56	5.5	11.9	251	27.1	1
carboplatin	M	75	Caucasian	adenocarcinoma	IV	Former	1	1.09	66	5.8	11.2	298	26.9	0
carboplatin	M	47	Caucasian	adenocarcinoma	IV	current	1	1.03	86	11.9	16.2	162	28.7	0
cisplatin	M	65	Caucasian	SCLC	IV	Current	2	0.63	103	6.5	8.8	246	23.4	0
carboplatin	M	64	Caucasian	adenocarcinoma	IV	Current	1	0.71	90	11.8	10.7	424	19.2	1
carboplatin	M	79	Caucasian	adenocarcinoma	IV	Never	1	0.86	82	12.4	13	440	28.8	0
carboplatin	M	58	other	adenocarcinoma	IV	Never	2	0.63	109	12.4	13	565	22.7	1
carboplatin	F	67	Caucasian	Squamous	II	Former	1	0.67	92	5.4	12	347	26.9	0
carboplatin	F	64	Caucasian	adenocarcinoma	IV	Former	1	0.69	92	8.1	12.8	320	27.8	0
cisplatin	F	54	Caucasian	adenocarcinoma	III	Never	0	0.92	71	5.5	14.3	327	19.0	0
carboplatin	F	63	Caucasian	adenocarcinoma	IV	Former	1	0.71	91	11.3	12.3	255	31.3	0
carboplatin	F	56	Caucasian	adenocarcinoma	IV	Current	1	1	63	8.3	11.6	391	17.0	0
carboplatin	F	75	Caucasian	adenocarcinoma	II	Current	1	0.68	86	7.4	9.3	199	29.4	0
carboplatin	F	68	Caucasian	SCLC	IV	Current	2	0.45	103	8.8	10.3	390	23.4	0
carboplatin	F	64	Caucasian	Squamous	IV	Former	1	0.86	71	18.9	13.7	374	29.6	0
carboplatin	F	73	Caucasian	SCLC	III	Current	2	0.71	85	8.2	11.9	295	18.5	0
carboplatin	F	52	Caucasian	SCLC	III	Current	1	0.56	107	7.9	9.3	265	28.8	0
carboplatin	F	86	Caucasian	adenocarcinoma	IV	Former	2	1.46	32	10.8	11.5	192	22.7	0
carboplatin	F	43	Caucasian	adenocarcinoma	IV	Never	1	0.47	121	5.1	12.5	196	20.5	0
carboplatin	M	62	Caucasian	other	IV	Former	2	0.74	98	10.5	14.8	202	40.1	1
carboplatin	M	64	Caucasian	other	IV	Current	1	1.07	73	17.4	15.2	246	32.8	1
carboplatin	F	56	Caucasian	other	IV	Current	0	0.61	102	7.8	13.6	200	31.6	0
cisplatin	F	66	Caucasian	Squamous	II	Former	1	0.65	92	9.9	13.9	285	32.3	0
carboplatin	F	62	Caucasian	adenocarcinoma	IV	Current	1	0.42	110	13.3	14.1	181	23.6	0
cisplatin	F	69	Caucasian	SCLC	I	Current	1	0.57	95	4.7	11.2	335	19.6	0
carboplatin	M	64	Caucasian	other	IV	Former	3	1.27	59	8.7	14.2	263	33.0	0
carboplatin	M	71	other	adenocarcinoma	IV	Never	1	0.82	89	9	14.4	311	22.5	0
carboplatin	M	61	AA	SCLC	IV	Current	1	1.37	63	5.9	14.2	205	29.7	0
carboplatin	F	56	Caucasian	SCLC	IV	Current	0	0.66	98	7.9	15.5	236	26.7	0
carboplatin	F	69	Caucasian	adenocarcinoma	IV	Former	1	0.77	78	7.7	12	359	24.2	0
carboplatin	F	69	Caucasian	SCLC	IV	Current	2	0.85	70	17.4	8	170	38.3	0
carboplatin	F	44	Caucasian	other	III	Current	1	0.65	105	10.4	13.5	179	25.1	0
cisplatin	M	56	Caucasian	Squamous	III	Current	2	0.65	108	6.8	12.6	385	32.1	0
carboplatin	M	66	Caucasian	Squamous	II	Former	1	0.8	93	8.4	13.2	230	24.9	0
carboplatin	F	69	Caucasian	Squamous	III	Current	1	0.57	94	10.6	14.5	396	23.4	0
carboplatin	F	70	Caucasian	other	IV	Current	1	1.1	49	8.4	13	255	30.8	1
carboplatin	M	54	Caucasian	SCLC	IV	Former	0	0.9	96	9.2	14	377	28.5	0
carboplatin	M	48	AA	adenocarcinoma	II	Current	2	0.75	125	13.8	13.3	172	23.9	0
carboplatin	F	69	Caucasian	adenocarcinoma	IV	Former	1	1.07	51	5.6	13.3	163	34.0	0
carboplatin	m	66	Caucasian	adenocarcinoma	IV	Current	1	0.78	94	12.6	12.2	258	27.1	0
carboplatin	M	65	Caucasian	Squamous	IV	Current	1	0.86	92	11.2	12.2	354	19.2	0
carboplatin	M	64	Caucasian	adenocarcinoma	IV	Current	3	0.97	82	8.6	17.4	178	25.6	0

carboplatin	F	50	Caucasian	Squamous	III	Current	1	0.63	104	7.5	12.9	280	30.5	1
cisplatin	M	57	AA	adenocarcinoma	IV	Former	1	0.66	123	11.2	9.7	582	24.8	0
carboplatin	M	60	AA	Squamous	IV	Current	2	0.88	104	9.7	11.9	354	24.7	0
cisplatin	M	41	Caucasian	adenocarcinoma	III	Current	1	0.68	119	12.3	9.6	590	20.8	1
cisplatin	F	59	Caucasian	adenocarcinoma	III	Former	0	0.85	75	6.4	13.5	276	27.7	0
cisplatin	F	47	Caucasian	adenocarcinoma	IV	Current	0	0.5	115	9.1	12.8	391	27.1	0
cisplatin	M	73	Caucasian	SCLC	IV	Current	2	0.51	106	9.8	11.9	441	22.9	0
carboplatin	M	62	AA	Squamous	IV	Current	0	1.93	42	3.9	7.7	135	36.5	0
carboplatin	M	80	Caucasian	adenocarcinoma	III	Former	2	1.04	67	8.1	12	141	19.5	0
cisplatin	F	65	Caucasian	adenocarcinoma	IV	Current	1	0.77	81	6	12.7	310	16.0	0
carboplatin	M	55	Caucasian	adenocarcinoma	IV	Current	0	0.71	106	12.7	10.6	365	20.0	0
cisplatin	M	70	Caucasian	Squamous	III	Former	1	0.96	79	5	12.1	174	36.7	0
carboplatin	M	72	Caucasian	other	III	Current	1	0.79	90	11.2	11.1	303	17.8	0
carboplatin	F	48	Caucasian	adenocarcinoma	IV	Never	1	0.67	104	11.8	10.7	207	28.6	1
carboplatin	M	70	AA	Squamous	III	Former	1	0.61	117	5.1	13	182	23.2	0
carboplatin	M	74	Caucasian	Squamous	III	Former	0	0.9	84	9.1	14.1	258	28.7	0
carboplatin	M	73	Caucasian	adenocarcinoma	IV	Former	1	0.65	97	6.5	13.9	248	25.1	0
carboplatin	F	64	Caucasian	Squamous	II	Current	1	1.06	55	8.9	13.1	229	23.3	0
carboplatin	F	71	Caucasian	Squamous	II	Former	0	0.7	50	4.3	11	208	24.5	0
carboplatin	M	69	Caucasian	SCLC	IV	Current	2	1.11	67	12.7	15.4	283	27.3	0
carboplatin	M	72	Caucasian	Squamous	III	Current	1	0.98	77	10.3	10.4	352	22.0	0
carboplatin	M	83	Caucasian	other	IV	Current	0	0.79	83	10.7	15	203	18.8	0
carboplatin	F	72	Caucasian	adenocarcinoma	II	Never	0	0.67	88	7.5	14.8	222	27.6	0
cisplatin	F	51	Caucasian	SCLC	IV	Former	3	0.68	102	20.9	13.9	184	35.2	0
carboplatin	F	80	Caucasian	SCLC	IV	Current	3	0.63	79	13.4	13.1	210	17.6	0
cisplatin	M	57	Caucasian	adenocarcinoma	III	Current	0	0.5	120	5.5	14.8	248	15.7	0
carboplatin	M	60	AA	other	IV	Current	1	0.51	134	17.6	9.6	270	21.2	1
carboplatin	F	68	Caucasian	SCLC	IV	Former	1	0.9	66	7.2	10.7	306	24.9	0
cisplatin	M	66	Caucasian	SCLC	IV	Former	0	0.75	95	9.2	12.5	184	28.1	0
cisplatin	F	63	Caucasian	adenocarcinoma	IV	Former	1	0.74	86	10.6	11	350	33.5	1
carboplatin	F	72	Caucasian	SCLC	IV	Former	1	0.83	71	17.2	15.1	262	28.9	1
carboplatin	M	82	Caucasian	adenocarcinoma	IV	Former	1	1	70	11.2	10	412	26.3	0
carboplatin	F	71	Caucasian	SCLC	IV	Former	0	0.71	86	4.7	9.1	296	19.6	0
carboplatin	M	58	Caucasian	Squamous	IV	Former	2	0.68	99	13.3	11	597	21.2	1
carboplatin	F	50	Caucasian	adenocarcinoma	IV	Current	1	0.41	120	16.8	9.7	709	26.1	0
carboplatin	M	73	Caucasian	other	I	Former	0	0.93	81	7.1	4.8	316	27.9	0
cisplatin	M	50	Caucasian	adenocarcinoma	IV	Former	0	0.8	102	4.5	14.8	156	29.0	1
cisplatin	F	71	Caucasian	adenocarcinoma	IV	Never	1	0.72	84	11.8	13.4	268	26.1	0
carboplatin	M	90	Caucasian	SCLC	II	Former	2	0.82	78	7.5	15.1	140	26.6	0
carboplatin	M	57	Caucasian	adenocarcinoma	IV	Current	2	0.58	112	16.3	14	366	21.3	0
carboplatin	M	55	Caucasian	adenocarcinoma	IV	Current	3	0.58	115	4.1	11.8	189	19.9	0
cisplatin	M	58	Caucasian	adenocarcinoma	II	Former	1	0.76	100	9.6	14.6	413	25.5	0
carboplatin	F	64	Caucasian	SCLC	IV	Current	2	0.38	112	5.3	11.5	342	20.4	0
carboplatin	M	72	Caucasian	adenocarcinoma	IV	Former	1	1.16	62	7.3	12.2	394	18.0	0
carboplatin	F	61	Caucasian	adenocarcinoma	IV	Current	1	0.46	108	7.6	10.2	327	49.5	0
cisplatin	F	57	Caucasian	Squamous	II	Former	0	0.64	99	5.4	14.2	171	17.7	0
carboplatin	M	83	Caucasian	Squamous	III	Former	1	0.93	76	19.4	12.9	423	24.2	0
carboplatin	M	51	AA	adenocarcinoma	IV	Never	1	0.89	114	4.4	14.3	332	24.2	0
carboplatin	F	52	Caucasian	adenocarcinoma	IV	Current	2	0.83	81	12.8	10	302	39.5	0
carboplatin	M	58	Caucasian	adenocarcinoma	IV	Current	0	0.84	96	9.7	14.5	422	27.5	0
carboplatin	F	71	Caucasian	SCLC	IV	Former	1	0.63	90	6.9	13.3	190	29.4	0
carboplatin	F	66	Caucasian	SCLC	IV	Former	1	0.54	98	12.8	12.2	284	33.3	1
carboplatin	M	67	Caucasian	adenocarcinoma	IV	Former	1	0.71	97	8.6	12.3	384	27.5	0
carboplatin	F	53	Caucasian	adenocarcinoma	IV	Current	1	0.61	103	7.5	13.5	353	19.8	0
carboplatin	M	72	Caucasian	SCLC	IV	Former	2	1.51	45	9.9	11.1	198	28.1	0
carboplatin	F	64	Caucasian	adenocarcinoma	IV	Former	1	0.7	90	6.8	15.1	307	32.3	0
carboplatin	M	81	Caucasian	Squamous	IV	Current	1	0.73	86	8.3	12.9	170	25.2	0
cisplatin	M	74	Caucasian	SCLC	IV	Current	1	0.91	83	7.1	14.2	113	15.9	0
carboplatin	M	74	Caucasian	Squamous	III	Former	1	0.86	85	9	11.5	357	22.4	1
carboplatin	F	71	Caucasian	adenocarcinoma	IV	Current	1	0.78	77	17.9	11.2	266	23.9	0
carboplatin	M	66	Caucasian	adenocarcinoma	I	Never	0	0.88	90	8.1	12.5	230	24.9	0
carboplatin	F	66	Caucasian	adenocarcinoma	III	Former	1	0.66	92	10.4	11	425	29.3	0
cisplatin	F	54	Caucasian	adenocarcinoma	II	Former	1	0.64	102	9.2	10.8	521	29.2	1
carboplatin	M	70	Caucasian	Squamous	III	Former	1	1.21	60	5.4	11.8	215	28.8	0
carboplatin	M	78	Caucasian	adenocarcinoma	IV	Former	1	0.7	90	6.4	10.9	332	25.1	0
carboplatin	F	53	Caucasian	adenocarcinoma	IV	Current	0	0.53	110	9.3	13.8	257	21.6	1
carboplatin	M	61	Caucasian	Squamous	IV	Current	2	0.72	100	3.3	12.7	185	25.8	0
carboplatin	M	69	Caucasian	adenocarcinoma	IV	Current	2	0.74	94	8	14.8	361	23.9	0
carboplatin	M	61	Caucasian	squamous	III	current	1	0.83	95	8.3	13.9	269	32.8	0
carboplatin	M	38	other	adenocarcinoma	IV	Current	1	0.63	125	10.2	12.1	318	23.4	0
carboplatin	M	62	Caucasian	adenocarcinoma	IV	Former	1	0.82	95	10.4	15.2	256	27.2	0
carboplatin	M	63	AA	other	IV	Current	1	0.74	113	12.5	14.1	376	15.9	0
cisplatin	F	56	Caucasian	SCLC	III	Current	1	0.89	73	8.7	11.1	206	23.4	0
carboplatin	M	58	Caucasian	squamous	III	former	2	0.86	95	10.3	13.1	185	38.3	0

carboplatin	F	69	Caucasian	Squamous	IV	Former	0	0.83	72	12.6	10	543	27.5	0
carboplatin	F	62	Caucasian	other	III	Former	1	1.07	56	8.6	15.2	280	17.2	0
carboplatin	M	57	Caucasian	adenocarcinoma	IV	Former	1	0.89	95	7.9	13.4	185	28.0	1
cisplatin	M	50	Caucasian	Squamous	III	Former	1	0.79	104	8.3	15.1	272	40.8	1
carboplatin	M	71	Caucasian	adenocarcinoma	II	Fromer	1	0.94	81	11.8	10.6	257	38.9	0
carboplatin	M	51	Caucasian	adenocarcinoma	IV	Current	1	1.13	74	11.2	13.9	283	30.5	1
carboplatin	F	53	Caucasian	Squamous	IV	Current	1	0.64	102	14	10.7	401	29.8	0
carboplatin	F	76	Caucasian	adenocarcinoma	IV	Former	1	1.14	47	6.9	12.4	229	20.8	0
cisplatin	F	65	Caucasian	adenocarcinoma	II	Former	0	0.69	100	4.8	12.8	301	28.8	0
carboplatin	M	68	Caucasian	SCLC	II	Former	2	1.5	49	6.2	8.4	261	26.7	0
carboplatin	M	37	Caucasian	adenocarcinoma	IV	Current	1	0.69	121	11.3	14.6	431	22.1	0
carboplatin	F	61	Caucasian	adenocarcinoma	IV	Former	1	0.61	98	7.2	11.1	356	26.2	0
carboplatin	F	68	Caucasian	adenocarcinoma	III	Former	3	0.87	69	12.1	13	262	47.3	0
carboplatin	F	86	Caucasian	adenocarcinoma	III	Former	1	0.72	76	5.3	13.1	303	21.3	0
carboplatin	F	67	Caucasian	other	IV	Never	2	0.51	99	9.7	13.9	275	23.9	0
carboplatin	F	55	Caucasian	Squamous	IV	Former	1	0.54	105	4.5	7.2	334	28.7	0
cisplatin	F	54	Caucasian	SCLC	IV	Current	1	0.36	122	10	13.4	301	23.6	0
carboplatin	F	62	Caucasian	Squamous	IV	Current	1	0.6	99	9.9	12.6	223	27.5	1
carboplatin	M	66	Caucasian	Squamous	II	Current	1	0.88	90	8.7	12.3	236	34.6	0
cisplatin	M	45	AA	adenocarcinoma	III	Current	1	0.84	116	7.4	14.9	280	17.8	0
carboplatin	M	51	Caucasian	adenocarcinoma	IV	Current	3	0.9	113	7.9	14.2	327	24.0	0
cisplatin	M	62	Caucasian	adenocarcinoma	III	former	0	0.83	109	9.6	13.6	403	27.3	1
carboplatin	F	47	Caucasian	adenocarcinoma	III	Current	1	0.5	116	9.1	11.6	414	28.6	0
carboplatin	M	66	AA	adenocarcinoma	IV	Current	0	0.98	92	9.9	13.9	248	22.5	0
carboplatin	F	68	Caucasian	Squamous	IV	Former	1	0.6	94	11.3	11.8	450	29.0	0
carboplatin	M	48	Caucasian	adenocarcinoma	IV	Current	1	0.56	122	15.9	15.2	346	21.3	1
cisplatin	M	32	Caucasian	SCLC	IV	Current	1	0.74	121	7	13.4	250	48.3	0
carboplatin	M	47	Caucasian	SCLC	IV	Current	0	0.76	109	15.3	15.7	419	24.6	0
carboplatin	M	77	Caucasian	Squamous	III	Former	0	1.04	68	7.2	12.2	285	32.1	0
carboplatin	F	79	Caucasian	Squamous	IV	Former	1	0.85	65	7.8	12.6	284	25.9	0
cisplatin	M	70	Caucasian	Squamous	III	Former	1	0.58	103	15	12	505	25.4	1
carboplatin	M	65	Caucasian	adenocarcinoma	III	Current	1	0.69	99	3.9	11.1	357	31.1	0
carboplatin	F	48	Caucasian	SCLC	IV	Current	2	0.79	89	9.5	9.4	378	28.9	1
carboplatin	F	81	Caucasian	adenocarcinoma	IV	Never	1	0.86	63	9	12.9	314	20.4	0
carboplatin	M	65	Caucasian	Squamous	III	Fromer	1	1.09	71	10.1	13.4	366	25.6	0
carboplatin	M	69	Caucasian	Squamous	IV	Former	1	1.35	53	8.1	15.3	221	27.3	1
carboplatin	M	70	Caucasian	SCLC	IV	Current	1	0.99	76	9.1	13.6	193	32.2	0
carboplatin	F	50	Caucasian	adenocarcinoma	IV	Current	1	0.61	105	8.6	13.8	269	22.9	1
carboplatin	F	69	AA	adenocarcinoma	IV	Never	1	0.88	78	6.4	13.7	328	29.2	1
carboplatin	M	65	Caucasian	adenocarcinoma	IV	Current	1	0.9	89	6.8	13.7	225	21.6	0
carboplatin	F	73	Caucasian	adenocarcinoma	IV	former	0	0.84	69	6.7	14.3	319	24.1	0
cisplatin	F	55	Caucasian	SCLC	IV	Current	1	0.77	86	8.9	11.2	363	22.5	1
carboplatin	M	51	Caucasian	adenocarcinoma	III	Current	0	0.77	104	10.2	14.1	354	27.1	0
cisplatin	M	55	Caucasian	squamous	III	Current	0	0.96	89	8.6	12	279	21.4	1
carboplatin	M	71	Caucasian	adenocarcinoma	II	former	1	1.08	68	11	15.5	432	26.2	1
carboplatin	M	69	Caucasian	other	III	Current	1	0.82	90	13.1	11.9	514	24.1	1
carboplatin	M	65	Caucasian	adenocarcinoma	III	current	1	0.9	89	15.3	12.1	367	24.4	0
carboplatin	F	69	Caucasian	Squamous	IV	former	1	0.84	71	7	12.5	450	27.0	0
cisplatin	M	53	Caucasian	adenocarcinoma	IV	former	0	0.79	103	13.3	12.6	445	25.0	1
carboplatin	F	76	Caucasian	adenocarcinoma	IV	Former	1	0.93	60	6.1	12.6	248	23.2	0
carboplatin	M	58	Caucasian	Squamous	IV	Current	0	0.95	88	8.2	14.8	234	22.2	0
carboplatin	F	74	Caucasian	SCLC	IV	Current	3	0.47	95	22.3	11.1	242	18.9	1
carboplatin	M	58	Caucasian	adenocarcinoma	II	Former	1	1.07	76	7.8	13.4	207	31.6	0
cisplatin	F	75	Caucasian	adenocarcinoma	III	Current	2	0.84	83	11.3	12.5	283	38.9	1
carboplatin	F	53	Caucasian	adenocarcinoma	IV	Former	1	1.01	57	12.8	9.9	511	24.6	0
carboplatin	M	60	Caucasian	adenocarcinoma	III	Former	1	0.98	83	6.8	15.6	146	27.8	0
cisplatin	M	64	Caucasian	SCLC	III	Current	0	0.64	103	11.2	16.8	326	27.6	0
carboplatin	M	64	Caucasian	adenocarcinoma	IV	Current	1	0.66	100	9.7	13.6	373	24.1	0
cisplatin	F	51	Caucasian	SCLC	IV	Current	2	0.56	108	5.5	12.9	345	23.7	1
cisplatin	M	63	Caucasian	SCLC	IV	former	0	0.83	85	8.3	15.7	300	24.5	0
carboplatin	M	75	Caucasian	adenocarcinoma	IV	Former	0	1.21	58	15.4	13	184	26.4	0
carboplatin	M	72	Caucasian	adenocarcinoma	IV	Former	1	0.84	87	3.9	9.3	122	25.6	1
cisplatin	M	52	Caucasian	SCLC	II	current	0	1.11	77	6.3	14.3	160	34.7	0
carboplatin	F	69	Caucasian	adenocarcinoma	II	Former	0	0.75	81	10.4	11.3	453	24.6	0
carboplatin	M	71	Caucasian	SCLC	IV	Current	3	1.07	68	6.3	9.9	165	27.2	0
carboplatin	M	67	Caucasian	adenocarcinoma	III	former	1	0.72	90	11.1	13.4	273	21.7	0
cisplatin	F	57	Caucasian	adenocarcinoma	III	current	0	0.69	97	12.8	13.1	269	21.5	0
carboplatin	M	65	Caucasian	squamous	III	former	1	0.76	94	10.3	14.5	294	34.9	0
cisplatin	M	55	Caucasian	Squamous	II	Current	0	0.93	92	5.3	13	470	22.9	0
carboplatin	M	53	Caucasian	SCLC	IV	Current	1	0.78	99	10.8	14.8	336	27.9	0
carboplatin	M	70	Caucasian	adenocarcinoma	IV	Current	1	0.85	88	8.8	13.5	200	22.5	0
carboplatin	F	70	Caucasian	adenocarcinoma	IV	former	1	0.6	92	7.3	11	324	26.3	1
carboplatin	M	61	Caucasian	other	IV	Current	1	0.84	93	6.6	14.4	271	27.6	0
carboplatin	F	56	Caucasian	other	IV	Current	1	0.72	94	13	14.7	296	24.6	0

carboplatin	M	80	Caucasian	adenocarcinoma	III	Former	1	0.93	77	5.5	14.4	215	24.1	1
carboplatin	M	68	Caucasian	SCLC	IV	Former	1	0.59	101	7.4	14.5	337	27.8	0
cisplatin	F	48	Caucasian	adenocarcinoma	IV	current	1	0.5	112	19.1	12.5	315	23.7	0
carboplatin	M	78	Caucasian	SCLC	IV	Current	0	0.55	111	11	10.7	169	19.9	0
carboplatin	F	80	Caucasian	Squamous	IV	never	1	0.8	70	8.1	11.5	312	24.3	1
cisplatin	M	63	Caucasian	SCLC	IV	current	0	0.85	92	8.1	14.2	211	28.2	0
carboplatin	F	57	Caucasian	adenocarcinoma	IV	Former	1	0.81	79	6.1	10	100	25.2	0
carboplatin	M	51	Caucasian	Squamous	III	Former	1	0.78	102	25	10.8	547	23.9	0
carboplatin	F	74	Caucasian	SCLC	IV	Current	3	1.01	55	11.3	14.8	135	28.0	0
carboplatin	F	66	Caucasian	SCLC	IV	Current	1	0.77	81	7	13.7	279	42.7	0
carboplatin	M	56	AA	adenocarcinoma	IV	current	0	1.17	78	6	14.5	247	23.2	0
cisplatin	F	53	Caucasian	SCLC	III	current	0	0.67	100	11	15.1	200	27.7	0
carboplatin	F	41	Caucasian	adenocarcinoma	IV	never	0	0.79	91	6.8	10.6	376	27.6	0
carboplatin	M	68	Caucasian	Squamous	III	Former	1	0.88	86	7	14	216	33.2	0
cisplatin	F	57	Caucasian	SCLC	IV	former	0	0.89	72	5.2	10.3	145	25.6	0
carboplatin	M	76	Caucasian	adenocarcinoma	IV	Former	1	0.9	89	11.6	13.5	482	27.5	0
carboplatin	M	61	AA	adenocarcinoma	IV	current	1	0.72	100	3.6	10	210	20.1	0
carboplatin	M	62	AA	Squamous	IV	Current	0	1.44	60	11.7	13.9	379	31.4	0
carboplatin	M	68	Caucasian	adenocarcinoma	II	never	1	0.84	90	3.7	12.9	169	29.9	0
cisplatin	M	57	Caucasian	SCLC	IV	Former	1	0.78	100	12.7	13.2	284	25.5	0
cisplatin	M	42	AA	Squamous	IV	current	1	1.04	88	7.7	9.9	556	22.5	0
cisplatin	F	65	Caucasian	SCLC	IV	current	1	0.52	99	10.9	10.6	595	19.0	0
cisplatin	M	69	Caucasian	adenocarcinoma	II	Current	1	0.9	87	2.2	15.2	96	26.2	0
carboplatin	M	67	Caucasian	other	IV	Current	2	0.98	77	14.1	14.3	248	37.1	0
cisplatin	F	59	Caucasian	adenocarcinoma	IV	Former	0	0.55	102	4.8	10	340	23.0	1
carboplatin	F	69	Caucasian	adenocarcinoma	III	Current	2	0.69	86	8.7	14.1	334	24.4	0
cisplatin	F	68	Caucasian	adenocarcinoma	III	Current	0	0.79	76	8.4	13.3	490	21.6	0
cisplatin	M	65	Caucasian	squamous	I	former	0	0.84	89	12.2	14.4	292	31.4	0
carboplatin	F	54	Caucasian	SCLC	IV	current	1	0.75	91	6.9	13.3	195	22.3	0
carboplatin	M	55	Caucasian	adenocarcinoma	III	current	1	0.84	96	7.4	13.7	316	21.3	0
carboplatin	F	73	Caucasian	adenocarcinoma	IV	never	1	0.77	75	6.9	13.5	308	24.0	0
carboplatin	M	72	Caucasian	squamous	IV	current	1	1.16	62	8.3	12.3	346	20.8	0
carboplatin	F	66	Caucasian	squamous	III	former	1	0.81	74	7.3	11.1	346	23.8	0
carboplatin	F	56	AA	adenocarcinoma	IV	Current	1	0.98	75	5.4	10.1	284	28.5	0
carboplatin	M	67	Caucasian	other	III	former	2	1	89	7.6	10.4	549	31.2	1
carboplatin	M	72	Caucasian	adenocarcinoma	IV	former	0	1.18	87	5.6	10.9	164	25.6	0
carboplatin	F	63	Caucasian	adenocarcinoma	IV	former	1	0.58	96	11.7	14.6	359	40.0	0
cisplatin	F	68	Caucasian	adenocarcinoma	IV	never	1	0.89	66	5.4	14.4	208	31.4	1
carboplatin	M	76	AA	Squamous	IV	former	0	0.66	100	4.7	11.9	182	23.6	0
cisplatin	M	54	Caucasian	Squamous	III	former	0	0.73	105	9.4	13.5	365	26.7	0
cisplatin	F	57	Caucasian	adenocarcinoma	III	former	0	0.72	99	13.7	12.8	282	17.7	0
carboplatin	M	78	Caucasian	adenocarcinoma	III	Never	1	0.95	75	5.9	10.2	264	28.5	0
carboplatin	F	76	Caucasian	adenocarcinoma	IV	former	1	0.73	80	9.4	13.7	341	33.1	0
carboplatin	F	60	Caucasian	adenocarcinoma	IV	never	1	0.75	86	9.9	12.1	166	28.0	0
carboplatin	F	75	Caucasian	adenocarcinoma	III	former	1	0.7	85	8.9	12.4	310	32.3	0
carboplatin	F	52	Caucasian	adenocarcinoma	IV	current	0	0.71	96	9.7	13.1	486	22.2	0
carboplatin	F	61	Caucasian	SCLC	IV	current	1	0.83	76	10.3	11.4	428	52.5	0
carboplatin	M	52	Caucasian	adenocarcinoma	IV	former	1	1	86	10.4	13.4	129	28.1	0
cisplatin	M	60	Caucasian	adenocarcinoma	III	current	0	0.7	105	11.6	11.7	582	21.6	0
carboplatin	M	54	other	adenocarcinoma	III	current	2	0.58	112	7.8	10.2	372	22.9	1
carboplatin	F	77	Caucasian	squamous	IV	current	2	0.79	72	19.4	10.3	378	22.0	1
carboplatin	F	61	Caucasian	adenocarcinoma	IV	current	2	0.63	94	8.8	14	351	23.9	0
cisplatin	M	65	Caucasian	SCLC	IV	current	1	0.66	105	7.1	15.2	258	16.5	0
carboplatin	M	63	Caucasian	adenocarcinoma	IV	former	2	0.7	88	8.1	12.6	290	20.0	0
carboplatin	F	67	Caucasian	SCLC	IV	current	1	2.51	20	8.8	12.2	342	27.3	0
carboplatin	M	60	Caucasian	squamous	II	current	1	1.06	76	9.5	14.8	272	18.8	0
carboplatin	M	68	Caucasian	SCLC	IV	current	3	1.17	62	7.4	8.5	223	32.0	0
carboplatin	M	71	Caucasian	adenocarcinoma	IV	former	1	1.02	76	6.6	12.4	271	33.5	0
carboplatin	M	80	Caucasian	SCLC	IV	former	3	1.17	63	11.4	13.8	250	32.4	0
carboplatin	M	80	Caucasian	SCLC	III	current	2	1.12	60	9.7	15.5	344	25.2	0
cisplatin	F	65	Caucasian	SCLC	IV	current	3	0.51	101	5.4	11.4	337	16.5	1
carboplatin	F	57	Caucasian	adenocarcinoma	III	former	0	0.92	67	5.4	12.1	301	27.5	0
carboplatin	M	63	Caucasian	squamous	IV	former	1	0.95	82	5	14.6	221	32.1	1
carboplatin	M	57	Caucasian	SCLC	IV	current	2	1.38	44	7.8	11.5	341	28.6	1
carboplatin	F	41	Caucasian	adenocarcinoma	IV	former	3	0.6	110	7.9	11.8	261	24.0	1
cisplatin	F	57	AA	other	III	current	1	0.75	81	5.9	12	204	23.2	1
carboplatin	M	58	Caucasian	SCLC	IV	current	1	0.86	93	5.5	11.9	341	22.0	0
carboplatin	F	75	Caucasian	other	IV	never	2	0.75	77	7.2	10	448	25.2	0
carboplatin	M	57	Caucasian	adenocarcinoma	IV	never	2	0.85	95	13	13.3	282	30.8	1
carboplatin	F	55	Caucasian	other	IV	current	1	0.7	100	11.9	12.3	483	18.3	0
carboplatin	M	79	Caucasian	SCLC	IV	never	2	0.96	73	6.1	14.6	273	22.6	0
carboplatin	F	57	AA	adenocarcinoma	IV	current	1	0.68	94	13	13.3	184	21.7	0
carboplatin	F	71	Caucasian	adenocarcinoma	IV	current	0	0.64	92	12.5	14.5	444	21.9	0
carboplatin	F	67	Caucasian	adenocarcinoma	IV	former	1	0.59	93	5.8	11.2	618	26.2	0

cisplatin	M	63	other	SCLC	IV	current	3	0.77	80	12.4	13.3	260	25.7	0
carboplatin	F	72	Caucasian	adenocarcinoma	IV	current	1	0.94	59	8.9	10.6	285	28.1	0
cisplatin	F	53	Caucasian	squamous	III	current	1	0.62	100	7.6	11	375	19.1	0
cisplatin	F	65	AA	squamous	III	never	1	0.72	93	7.5	12.9	316	33.0	0
cisplatin	F	49	Caucasian	SCLC	IV	current	3	0.71	106	3.3	10.2	209	24.5	0
cisplatin	F	63	Caucasian	adenocarcinoma	III	former	0	0.81	86	7.3	13.4	315	22.1	0
carboplatin	M	55	Caucasian	adenocarcinoma	IV	current	1	0.71	108	21.6	15.5	246	23.9	0
carboplatin	M	56	Caucasian	adenocarcinoma	IV	never	0	1.09	71	8.6	13.2	299	23.7	0
carboplatin	M	84	Caucasian	squamous	III	current	1	0.75	84	12.2	13.7	225	23.6	0
carboplatin	F	59	Caucasian	adenocarcinoma	IV	current	0	0.73	88	17.8	13.6	311	29.6	0
carboplatin	M	56	Caucasian	adenocarcinoma	IV	current	2	0.73	101	8.2	14.7	413	22.1	0
cisplatin	M	59	AA	adenocarcinoma	II	current	2	0.96	98	6.2	12.4	224	21.4	0
carboplatin	M	70	Caucasian	squamous	III	current	2	0.9	86	8	15.2	282	30.0	1
carboplatin	F	75	Caucasian	SCLC	III	current	3	0.77	74	11	13.8	263	20.1	0
carboplatin	F	54	Caucasian	adenocarcinoma	II	current	1	0.77	86	6	12.3	177	28.1	1
carboplatin	F	64	AA	squamous	IV	Former	1	1.09	60	6.5	12.4	260	31.6	0
carboplatin	M	74	Caucasian	SCLC	IV	former	0	1.05	69	8	14	95	30.9	1
carboplatin	M	57	Caucasian	SCLC	IV	current	3	0.81	97	6.6	12	214	25.8	0
carboplatin	m	77	Caucasian	adenocarcinoma	IV	current	0	0.82	76	5.3	14.1	201	24.3	1
cisplatin	M	64	Caucasian	squamous	II	current	1	0.92	91	10.9	14.3	308	26.5	0
carboplatin	M	74	Caucasian	adenocarcinoma	IV	former	2	0.7	91	7.8	13.9	258	28.1	0
carboplatin	F	78	Caucasian	adenocarcinoma	III	former	2	0.87	63	7.6	13.1	213	31.3	1
carboplatin	F	55	Caucasian	other	IV	current	2	0.6	110	10.3	12.4	367	16.7	1
carboplatin	F	75	Caucasian	squamous	III	former	1	0.78	73	6.6	14.1	187	25.9	0
cisplatin	M	58	Caucasian	squamous	III	current	1	0.68	102	13	13.5	497	18.9	0
cisplatin	F	78	Caucasian	SCLC	III	never	1	0.49	92	3.3	11.8	270	22.6	0
cisplatin	F	55	Caucasian	adenocarcinoma	IV	never	2	0.66	98	6.4	12.4	206	32.8	1
carboplatin	M	48	AA	adenocarcinoma	III	former	1	1.11	89	4.9	14.6	210	41.1	1
carboplatin	F	47	AA	SCLC	III	current	1	0.79	100	6	10.8	626	26.2	0
carboplatin	M	67	Caucasian	adenocarcinoma	IV	current	2	1.11	74	7.3	10.5	180	27.6	0
carboplatin	M	58	AA	adenocarcinoma	IV	current	2	1.08	73	5.5	14.1	150	21.9	1
carboplatin	M	82	Caucasian	adenocarcinoma	III	former	2	1.15	57	5.6	9.9	154	25.0	0
cisplatin	M	66	Caucasian	adenocarcinoma	III	former	0	0.84	89	5.8	14.9	285	27.0	0
cisplatin	F	57	Caucasian	SCLC	IV	former	3	0.7	100	17.5	14.5	224	34.6	1
cisplatin	F	64	Caucasian	SCLC	IV	current	3	0.51	98	6.4	11.5	240	26.6	0
carboplatin	F	65	Caucasian	adenocarcinoma	IV	never	1	0.55	98	6.6	12.4	314	18.8	0
carboplatin	M	48	Caucasian	adenocarcinoma	III	current	1	0.69	110	9.5	11.6	372	33.3	0
carboplatin	M	81	Caucasian	squamous	IV	former	2	0.73	88	8.7	12.1	413	22.8	0
carboplatin	F	73	Caucasian	SCLC	III	former	2	0.82	67	7.9	10.9	38	34.9	0
cisplatin	M	53	Caucasian	squamous	III	current	1	0.71	104	6.6	12.4	290	40.7	0
carboplatin	F	49	other	adenocarcinoma	IV	never	0	0.79	103	4.9	12.3	283	18.7	0
cisplatin	F	58	Caucasian	SCLC	IV	current	2	0.6	100	8.7	15	142	21.3	0
carboplatin	F	73	Caucasian	adenocarcinoma	IV	former	1	0.42	99	8.7	10.7	293	18.9	0
carboplatin	M	81	Caucasian	SCLC	IV	current	2	0.73	75	7.5	15	190	25.6	0
carboplatin	M	50	Caucasian	squamous	IV	current	1	0.92	94	6.3	14.4	204	24.1	1
carboplatin	M	72	Caucasian	SCLC	I	current	2	1.78	41	17.4	12.6	277	23.7	0
carboplatin	M	67	AA	squamous	III	current	0	0.96	80	9	9.9	385	21.4	0
carboplatin	F	66	Caucasian	adenocarcinoma	IV	current	1	0.72	82	10.1	12.5	425	22.8	0
carboplatin	M	69	Caucasian	adenocarcinoma	III	current	0	0.82	89	7	13	254	25.9	0
carboplatin	F	58	Caucasian	SCLC	IV	current	2	0.72	91	10.3	11.2	351	24.4	0
carboplatin	M	58	Caucasian	squamous	III	current	2	1.39	55	10.1	11.1	536	22.7	0
cisplatin	F	67	Caucasian	SCLC	IV	current	1	0.57	96	14.3	12.3	406	17.3	0
carboplatin	M	59	Caucasian	adenocarcinoma	IV	current	2	0.82	95	11.8	16.6	373	21.6	0
carboplatin	F	42	Caucasian	adenocarcinoma	IV	former	2	0.69	108	5.9	13.4	322	22.4	0
cisplatin	M	67	Caucasian	other	III	current	3	0.69	96	9.9	13.8	283	27.3	0
carboplatin	M	59	Caucasian	adenocarcinoma	III	current	1	0.83	93	2.6	9.8	238	29.9	0
carboplatin	F	63	Caucasian	squamous	III	current	2	0.65	92	7.7	13.5	284	27.3	0
carboplatin	F	62	Caucasian	SCLC	IV	current	2	0.48	103	8.2	11.6	312	24.3	0
carboplatin	M	61	Caucasian	adenocarcinoma	IV	former	0	0.89	89	8.2	14	331	35.0	0
carboplatin	M	73	Caucasian	adenocarcinoma	III	former	1	0.92	88	8.2	14.4	305	25.3	0
cisplatin	M	47	other	squamous	IV	former	2	0.66	108	2.6	12.5	137	30.1	0
carboplatin	M	55	Caucasian	adenocarcinoma	IV	current	2	0.84	85	9.8	14.4	305	25.0	1
cisplatin	M	71	Caucasian	squamous	IV	former	1	0.93	89	6.7	13.2	355	26.9	0
carboplatin	M	80	Caucasian	adenocarcinoma	III	current	2	1.03	72	8	13.1	228	24.9	0
carboplatin	M	58	Caucasian	adenocarcinoma	IV	never	1	0.81	95	6.2	15.9	214	28.7	0
cisplatin	M	54	Caucasian	adenocarcinoma	III	current	2	0.79	100	9	15.1	314	24.8	0
carboplatin	M	79	Caucasian	adenocarcinoma	IV	former	2	1.2	56	30.1	10.1	144	20.7	0
cisplatin	F	69	Caucasian	SCLC	IV	former	1	0.51	96	3.5	8.7	167	29.0	0
carboplatin	F	79	Caucasian	adenocarcinoma	IV	never	1	0.57	88	15.7	14.8	184	31.3	0
cisplatin	F	73	Caucasian	adenocarcinoma	III	former	2	0.74	79	7.8	13	280	29.4	0
carboplatin	M	80	AA	other	IV	former	2	1.21	49	5.5	11.9	171	24.2	1
carboplatin	F	75	Caucasian	other	IV	current	3	0.47	96	8.4	12.7	305	23.6	1
carboplatin	F	79	Caucasian	other	I	former	0	0.74	75	5.8	11.6	335	19.3	1
carboplatin	M	51	Caucasian	Other	IV	current	1	0.93	93	19.3	11.5	134	23.5	1

carboplatin	F	79	Caucasian	squamous	IV	former	2	0.83	75	9.8	11.2	315	32.6	0
carboplatin	F	50	Caucasian	adenocarcinoma	IV	current	2	0.57	108	21.1	10.1	608	24.4	0
carboplatin	F	64	Caucasian	other	II	current	2	0.71	96	15.8	8	496	33.8	0
carboplatin	F	56	AA	adenocarcinoma	IV	current	1	0.88	79	3.8	14.3	186	31.0	0
cisplatin	F	35	Caucasian	SCLC	IV	never	3	0.69	113	15.8	12.7	522	41.5	0
carboplatin	M	60	AA	other	IV	current	2	0.62	120	12.1	11.7	312	30.0	0
carboplatin	F	57	AA	SCLC	IV	current	2	0.76	99	10.2	11.5	776	42.7	0
cisplatin	M	61	other	other	II	current	2	0.97	82	5.6	15.7	133	21.9	0
carboplatin	F	67	Caucasian	adenocarcinoma	IV	current	2	0.73	92	9.6	14.3	368	24.5	0
carboplatin	F	74	AA	adenocarcinoma	IV	Current	2	0.64	101	7.2	12.3	288	28.6	1
carboplatin	M	89	Caucasian	squamous	IV	former	1	0.87	76	6.1	12.7	188	29.2	0
carboplatin	M	73	Caucasian	adenocarcinoma	IV	current	2	0.83	86	8.1	12.9	387	22.9	0
cisplatin	M	59	Caucasian	squamous	III	former	2	0.81	101	8.5	14.3	297	29.5	0
cisplatin	M	60	Caucasian	SCLC	IV	current	0	0.81	95	16.7	15.5	210	38.0	1
carboplatin	F	79	Caucasian	adenocarcinoma	IV	former	2	0.95	58	7.7	12.1	231	18.4	0
carboplatin	M	58	Caucasian	adenocarcinoma	III	current	2	0.93	89	8.2	14.7	290	22.9	0
carboplatin	M	88	Caucasian	adenocarcinoma	III	never	1	1.38	45	6.4	12.9	199	19.9	0
carboplatin	F	52	Caucasian	adenocarcinoma	IV	current	1	0.72	91	17.8	11.5	214	32.9	1
cisplatin	M	66	Caucasian	adenocarcinoma	III	former	0	0.84	89	5.8	14.9	167	33.3	0
carboplatin	F	52	Caucasian	adenocarcinoma	IV	current	0	0.62	104	8.3	12.8	252	26.5	0
carboplatin	F	63	Caucasian	SCLC	IV	current	2	0.57	93	9.7	13.5	333	23.3	0
carboplatin	M	75	other	SCLC	IV	never	2	0.94	93	2.5	12.3	139	21.2	0
cisplatin	F	66	Caucasian	SCLC	IV	current	2	0.45	94	12.5	16.3	389	34.0	1
carboplatin	M	55	Caucasian	squamous	III	current	1	0.7	94	5.8	12.5	363	22.6	0
carboplatin	M	59	Caucasian	adenocarcinoma	IV	former	2	0.74	102	12.1	12	244	32.8	0
carboplatin	F	81	Caucasian	SCLC	III	former	3	0.58	84	7.1	12.1	494	24.1	0
carboplatin	F	54	Caucasian	SCLC	I	current	0	0.54	117	4.2	13.1	195	20.3	0
carboplatin	F	75	Caucasian	adenocarcinoma	IV	former	1	0.78	73	6.9	10.7	199	37.1	0
carboplatin	M	75	Caucasian	adenocarcinoma	III	former	2	1.21	44	8	11.7	273	31.5	0
carboplatin	F	71	Caucasian	squamous	IV	former	2	0.94	64	6.4	7.7	390	23.3	0
carboplatin	F	71	Caucasian	adenocarcinoma	III	former	1	0.81	74	8.8	12.7	294	33.6	0
carboplatin	M	65	AA	adenocarcinoma	IV	current	1	0.97	89	4.9	12.4	258	23.9	0
cisplatin	F	65	Caucasian	SCLC	III	current	1	0.67	98	4.5	11.5	263	31.9	1
carboplatin	M	74	Caucasian	squamous	IV	current	2	0.92	77	5.9	12.3	321	21.6	1
carboplatin	F	64	Caucasian	adenocarcinoma	IV	former	1	0.48	101	6.2	12.7	338	16.7	0
carboplatin	M	64	AA	SCLC	I	former	2	0.77	104	7.2	13.5	166	25.4	0
carboplatin	F	56	Caucasian	adenocarcinoma	IV	current	1	0.57	103	7.8	14.8	229	31.2	1
carboplatin	F	58	AA	SCLC	IV	former	2	1.32	44	11.2	11.3	245	41.0	0
carboplatin	M	64	Caucasian	adenocarcinoma	IV	current	0	0.82	91	6.4	12	128	22.0	0
carboplatin	M	73	Caucasian	SCLC	IV	former	1	0.78	67	6.1	10.8	175	29.6	0
cisplatin	F	56	AA	adenocarcinoma	III	never	2	0.84	76	8.4	12	327	22.1	0
carboplatin	F	56	Caucasian	SCLC	IV	current	2	0.72	94	14.2	13.3	138	33.6	0
carboplatin	M	61	Caucasian	SCLC	IV	current	2	0.69	94	6.5	11.4	327	24.3	0
cisplatin	M	61	Caucasian	SCLC	IV	current	1	0.77	80	16.6	9.6	323	18.4	0
cisplatin	M	63	Caucasian	SCLC	IV	former	2	0.74	98	10.7	14.5	225	35.8	0
carboplatin	M	61	Caucasian	squamous	IV	former	1	0.75	93	8.8	13.8	287	25.5	0
